# Regulation of the Central Carbon Metabolism in Apple Fruit Exposed to Postharvest Low-Oxygen Stress

**DOI:** 10.3389/fpls.2019.01384

**Published:** 2019-10-30

**Authors:** Jelena Boeckx, Suzane Pols, Maarten L. A. T. M. Hertog, Bart M. Nicolaï

**Affiliations:** ^1^KU Leuven, BIOSYST-MeBioS, Leuven, Belgium; ^2^Flanders Centre of Postharvest Technology, Leuven, Belgium

**Keywords:** low-oxygen stress, postharvest storage, apple fruit, central carbon metabolism, oxygen sensing

## Abstract

After harvest, fruit remain metabolically active and continue to ripen. The main goal of postharvest storage is to slow down the metabolic activity of the detached fruit. In many cases, this is accomplished by storing fruit at low temperature in combination with low oxygen (O_2_) and high carbon dioxide (CO_2_) partial pressures. However, altering the normal atmospheric conditions is not without any risk and can induce low-O_2_ stress. This review focuses on the central carbon metabolism of apple fruit during postharvest storage, both under normal O_2_ conditions and under low-O_2_ stress conditions. While the current review is focused on apple fruit, most research on the central carbon metabolism, low-O_2_ stress, and O_2_ sensing has been done on a range of different model plants (e.g., *Arabidopsis*, potato, rice, and maize) using various plant organs (e.g., seedlings, tubers, roots, and leaves). This review pulls together this information from the various sources into a coherent overview to facilitate the research on the central carbon metabolism in apple fruit exposed to postharvest low-O_2_ stress.

## Postharvest Storage of Apple Fruit

### Fruit Ripening

Fruit ripening is the process through which a fully grown mature but inedible plant organ is transformed into an attractive edible fruit with an optimum blend of colour, aroma, and texture. During this complex, highly coordinated and genetically programmed transformation process a sequence of changes occurs (Giovannoni, 2001), which is reflected by the metabolic activity of the fruit. Fruit can be divided into two groups based on their respiratory behavior during ripening: climacteric and nonclimacteric fruit (Saltveit, 1999; Wills and Golding, 2016). For climacteric fruit like pears and apples, the ripening process is characterized by a rise in respiratory activity and ethylene production. This respiratory climacteric rise marks the end of a period of active synthesis and maintenance and the start of fruit senescence. For nonclimacteric fruit, such as orange, lemon, pineapple, grapes, and strawberry, there is no peak in respiration and ethylene production rates remain low (Alexander and Grierson, 2002; Paul et al., 2012; Wills and Golding, 2016). To maximize their storage potential, apples are harvested 1 or 2 weeks before their climacteric rise in respiration. Subsequently, the mature but unripe fruit is typically stored under low O_2_ conditions retarding the metabolic activity of the fruit to delay fruit ripening and senescence.

### Factors Controlling Fruit Respiration Rate

Postharvest respiration rate can be controlled through temperature, O_2_ and CO_2_ concentration (Lammertyn et al., 2001; Cameron et al., 1994; Hertog et al., 1998). Ho et al. (2018) showed that temperature is the most influential factor controlling the respiration rate of apple fruit. The inhibitory effect of low temperature on the respiration rate is due to the decrease in catalytic activity of respiratory enzymes (Lyons, 1973; Graham and Patterson, 1982; Atkin et al., 2005; Bron et al., 2005; Falagán and Terry, 2018). Therefore, to minimize respiration, fruit is stored at the lowest possible temperature (Jackman et al., 1988; Wang, 2010).

**Figure 1:**
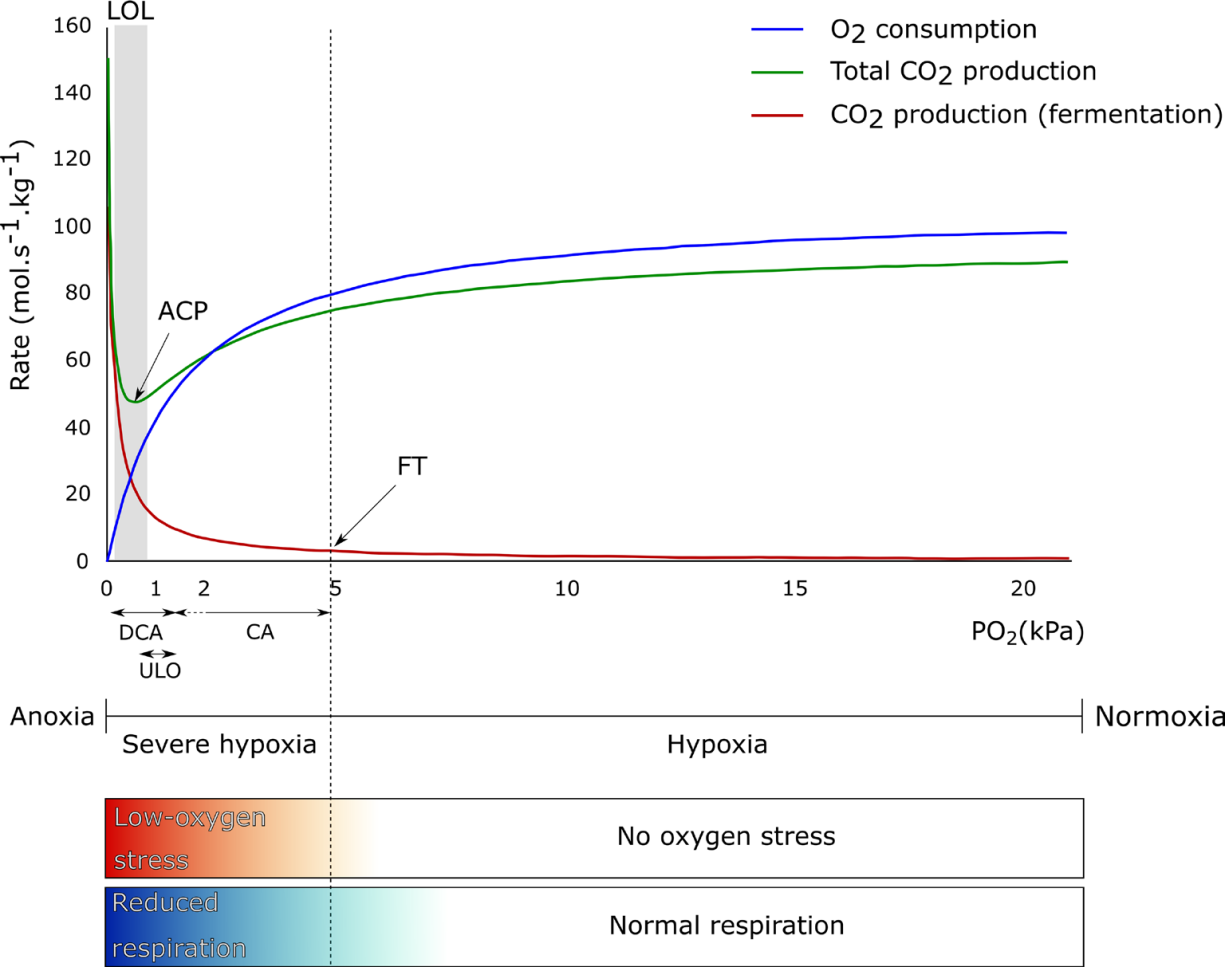
Theoretical effect of O_2_ concentration on O_2_ consumption (blue line), total CO_2_ production (green line), and CO_2_ production from fermentation (red line) by apple fruit. The anaerobic compensation point (ACP), fermentation threshold (FT), and lower O_2_ limit (LOL) are indicated, as well as the range of PO_2_ applied in controlled atmosphere (CA), ultralow O_2_ (ULO), and dynamic controlled atmosphere (DCA) storage. The term no O_2_ stress is used to define all O_2_ levels where enough O_2_ is available for the fruit to have a normal respiratory metabolism, ranging from normoxia (typically 20.6% O_2_ at 101.325 kPa and 20°C) and below (hypoxia). The term low-O_2_ stress is used when O_2_ levels are limited (severe hypoxia) or completely absent in the environment (anoxia) and the fruit needs to reduce or adapt their respiratory metabolism. The color gradients mirror the gradual transitions between no O_2_ stress and low-O_2_ stress and between normal respiration and reduced respiration.

Next to temperature, storage atmosphere composition has an influence on fruit respiration rate. O_2_ is the final electron acceptor in the electron transport chain. By lowering the O_2_ concentration in the storage atmosphere, the respiration of many fruit and vegetables slows down (Biale, 1946; Ke et al., 1991; Yearsley et al., 1996; Teixeira and Durigan, 2010; Thompson, 2010). The metabolic respiration chain consist of different pathways in which CO_2_ is produced (glycolysis and TCA cycle) and O_2_ is consumed (mitochondrial electron transport chain). As indicated in **Figure 1**, the respiration rate, represented by the O_2_ consumption rate (blue line) and the total CO_2_ production rate (green line) until the fermentation threshold (FT), decreases as the O_2_ level decreases. However, when the O_2_ level moves toward anoxia, the fruit metabolism shifts from aerobic respiration to fermentation (Geigenberger, 2003; Prange et al., 2005) (see also section on the regulation of central carbon metabolism under low-O_2_ stress conditions), resulting in flavor and storage disorders (Kader et al., 1989; Franck et al., 2007; Thompson, 2010). Due to this metabolic shift, the CO_2_ produced by the glycolysis will increase again, as well as the CO_2_ produced by fermentation (red line), leading to an increase in the total CO_2_ production (green line). The O_2_ concentration at which the total respiratory CO_2_ production rate is minimal is called the anaerobic compensation point (ACP) (Prange et al., 2005).

Finally, also the CO_2_ concentration can influence the respiration rate of the fruit, but its mode of action is still not well understood. In addition, the effect of CO_2_ depends on the fruit (Kubo et al., 1989; Peppelenbos and van’t Leven, 1996; Hertog et al., 1998).

### Low-O_2_ Storage of Fruit

Apple fruit is commonly stored under low O_2_ conditions at low temperature with the optimal conditions varying by geographic location, harvest date, storage duration, cultivar, and season (Saltveit, 2003; Dilley, 2010; Watkins and Nock, 2012). Commercial storage conditions are typically set at constant safe values with O_2_ levels kept above the lower O_2_ limit (LOL) below which the fruit starts developing disorders ( Yearsley et al., 1997; Yahia, 2009; Wright et al., 2011). Since the LOL may be slightly different every year, a safe but likely suboptimal O_2_ concentration above the ACP is often maintained at the cost of higher quality losses (Wright et al., 2011; Bessemans et al., 2016).

As the static storage approach does not always provide optimal poststorage results, dynamic low-O_2_ storage approaches have been developed where the O_2_ conditions are adapted to the changing fruit physiology (Veltman et al., 2003; Wright et al., 2012; Bessemans et al., 2016).

While there are some studies on the effect of long-term low-O_2_ storage on metabolic adaptations of apple fruit during low-O_2_ storage (Saquet and Streif, 2008; Bekele et al., 2015; Brizzolara et al., 2017), and physiological disorders, (Pedreschi et al., 2007; Pedreschi et al., 2008; Pedreschi et al., 2009; Vandendriessche et al., 2013; Hatoum et al., 2014; Mellidou et al., 2014) the precise mode of action of low O_2_ not is well known. Cukrov et al. (2016) have shown that “Granny Smith” apples stored at 0.4 or 0.8 kPa O_2_ have a clearly different metabolic and transcriptomic profile. Brizzolara et al. (2017) demonstrated that “Granny Smith” and “Red Delicious” apples respond differently to low-O_2_ storage, suggesting that the genetic background played a key role in determining and modulating the observed metabolic changes to changed O_2_ levels. Hence, to further optimize low-O_2_ storage, it is important to get a better understanding on the fruit’s central carbon metabolism and the regulatory mechanisms involved in the responses of apple fruit to hypoxic conditions.

## Central Carbon Metabolism of Apple Fruit

The following sections present data based on model species such as *Arabidopsis* and rice, which is extended with information specifically for apple fruit, whenever available.

### Central Carbon Metabolism in the Absence of O_2_ Stress Conditions

**Figure 2:**
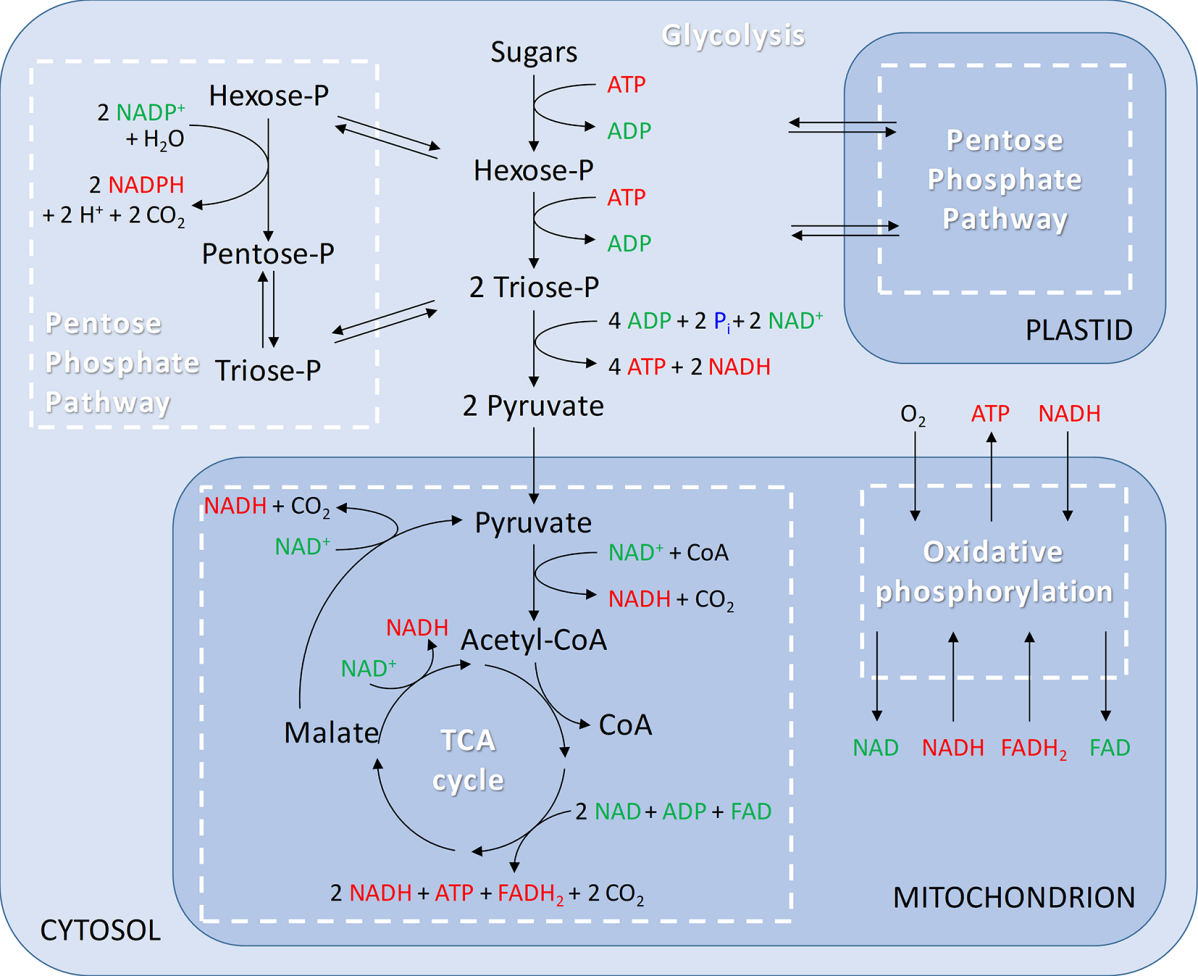
Generalised overview of respiration including glycolysis in the cytosol; pentose phosphate pathway in the cytosol and plastids; tricarboxylic acid (TCA) cycle and oxidative phosphorylation in the mitochondria.

When O_2_ is not limiting, apples produce their energy by completely oxidizing sugars through the respiratory metabolism consisting of the following pathways: glycolysis, pentose phosphate pathways (PPP), tricarboxylic acid (TCA) cycle, and the mitochondrial electron transport chain (mETC) (**Figure 2**). To appreciate their importance with regard to low-O_2_ storage, these pathways will first be summarized.

Glycolysis is an O_2_ independent pathway involving a series of enzymatic reactions that break down hexoses (mainly glucose and fructose) into pyruvate. To degrade sucrose *via* the glycolytic pathway, it is first cleaved by invertase (Bologa et al., 2003). The glycolysis consists of an energy consuming phase and an energy conserving phase. In the initial energy consuming phase, hexose (glucose or fructose) is phosphorylated requiring two molecules of adenosine triphosphate (ATP), and subsequently split into triose phosphates (i.e., glyceraldehyde 3-phosphate and dihydroxyacetone-phosphate). In the energy conserving phase, each triose phosphate is oxidized to pyruvate, providing two molecules of ATP and one molecule of reduced nicotinamide adenine dinucleotide (NADH). Overall, the glycolysis results in a net production of two molecules of ATP and two molecules of NADH, the major biochemical electron transporter and co-enzyme in plants. In plant cells, glycolysis can occur both in the cytosol and plastids (Plaxton, 1996; Mauseth, 2008; Taiz and Zeiger, 2010; António et al., 2016; Garrett and Grisham, 2017).

An alternative route for the cell to oxidize sugars is provided by the PPP, which takes place in the cytosol and in the plastids (**Figure 2**). The PPP is divided into an oxidative and a nonoxidative branch. The initial oxidative branch of the PPP oxidizes glucose 6-phosphate to ribulose 5-phosphate. In nonphotosynthetic cells, like apple cells, the oxidative branch of the PPP is a major source of reduced nicotinamide adenine dinucleotide phosphate (NADPH), which is used in biosynthetic processes such as fatty-acid synthesis and the assimilation of inorganic nitrogen (Neuhaus and Emes, 2000; Kruger and Von Schaewen, 2003). Furthermore, NADPH plays an important role in maintaining the redox potential necessary to protect plants against oxidative stress (Juhnke et al., 1996). Nowadays, the basic functions of the oxidative branch of the PPP are well-established (Mauseth, 2008; Taiz and Zeiger, 2010; Garrett and Grisham, 2017), but details of how the pathway operates in plants and how it influences other processes remain largely unknown. The reversible nonoxidative branch converts ribulose 5-phosphate to intermediates of the glycolysis, i.e., fructose 6-phosphate and glyceraldehyde 3-phosphate. This part of the PPP is a source of carbon skeletons for the synthesis of nucleotides, aromatic amino acids, phenylpropanoids, and their derivatives. Although both glycolysis and the PPP are involved in sugar oxidation in plants, the PPP only accounts for 15% to 30% of the hexose phosphate oxidized to glyceraldehyde 3-phosphate and CO_2_ (Kruger and Von Schaewen, 2003).

The next step in the respiratory metabolism is the TCA cycle, which takes place in the matrix of the mitochondria. Pyruvate produced in the glycolytic pathway is used as a substrate to fuel the TCA cycle and is transported across the inner membrane of the mitochondria by a mitochondrial pyruvate carrier (Li et al., 2014). Alternatively, pyruvate can be generated in the matrix from malate by the action of malic enzyme (Jacoby et al., 2012). Inside the mitochondria, pyruvate is oxidatively decarboxylated by pyruvate dehydrogenase complex (PDH) generating acetyl-CoA, CO_2_, and NADH (**Figure 2**). Acetyl-CoA is then incorporated in the TCA cycle by combining acetyl-CoA with oxaloacetate to form citrate. In the next steps, the two remaining carbon atoms of pyruvate are released as CO_2_. Besides the production of ATP, the TCA cycle also stores energy in the form of NADH and flavin adenine dinucleotide (FADH_2_). The intermediates of the TCA cycle also serve as substrates in the biosynthesis of amino acids, nucleic acids, and cell wall components needed for plant growth and development (Taiz and Zeiger, 2010; Harvey Millar et al., 2011; Jacoby et al., 2012).

**Figure 3:**
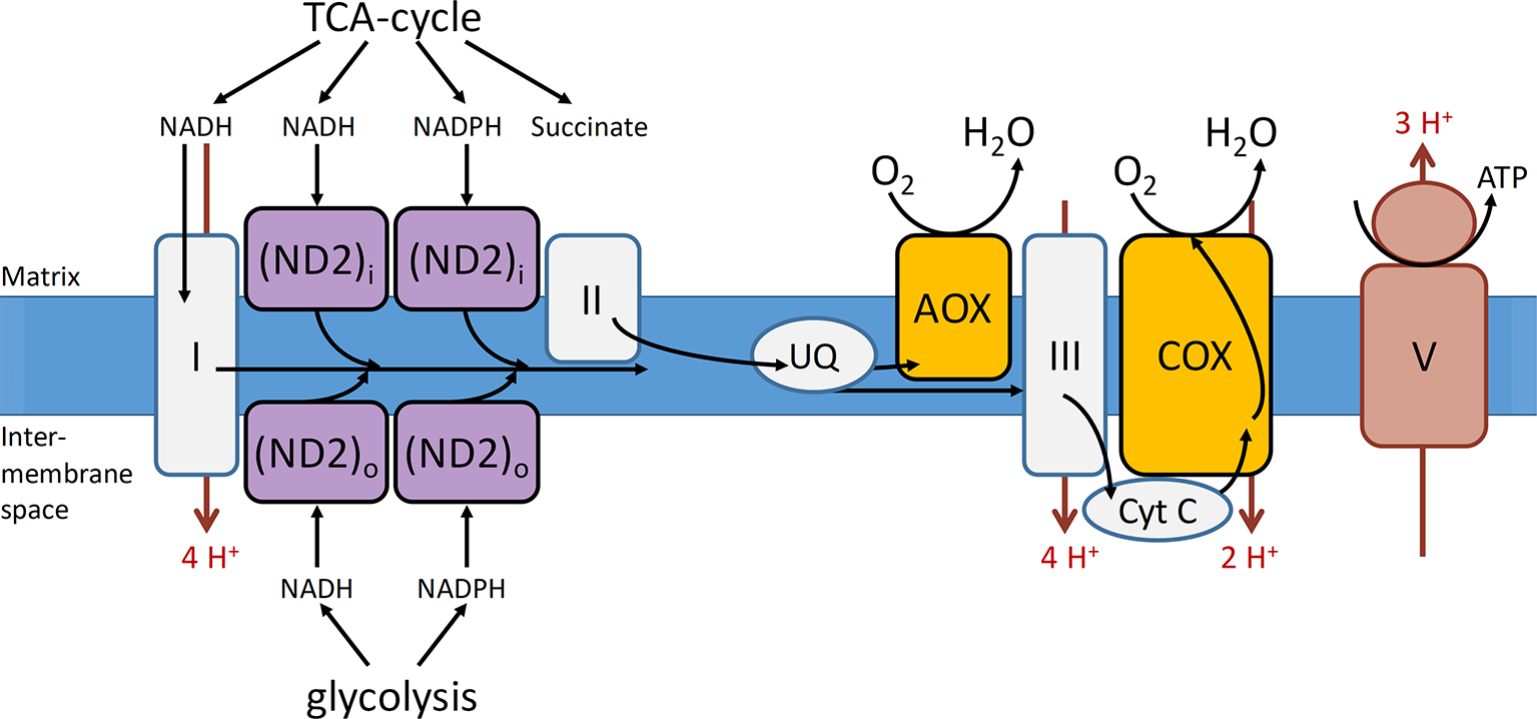
Representation of the mitochondrial electron transport chain with the conventional and alternative reactions located in the inner mitochondrial membrane. Complexes I–III, that is, NADH dehydrogenase, succinate dehydrogenase, cytochrome c reductase, respectively; COX, cytochrome c oxidase; V, ATP synthase; AOX, alternative oxidase; cyt c, cytochrome c; ND2i and ND2o, type II NAD(P)H dehydrogenase located at the inside (i) and outside (o) of the mitochondrial inner membrane; UQ, ubiquinone pool. The electron flow is indicated with black arrows and the proton flow with red arrows (reprinted with slight modification from Päpke et al., 2014. Copyright Springer-Verlag).

The mETC is where the final and only O_2_ consuming process of the respiratory metabolism, the oxidative phosphorylation, takes place (**Figure 2**). The mETC is located in the inner mitochondrial membrane and consist of several dehydrogenases, cytochrome oxidases, and an alternative oxidase (AOX). The reducing equivalents (NADH and FADH_2_) and succinate, produced inside the mitochondria in the TCA cycle, transfer their electrons to NADH dehydrogenase (complex I) and succinate dehydrogenase (complex II), respectively. The mitochondria of plants contain several additional nonphosphorylating NAD(P)H dehydrogenases (ND2) on the outside, as well as on the inside of the inner membrane (**Figure 3**). These enzymes enable plant mitochondria to oxidize NADH and NADPH, formed in the glycolysis and PPP, directly from the cytosol. The reduced compounds need to be oxidized to enable the respiratory metabolism to function continuously. All the dehydrogenases (complex I, complex II, and ND2) are linked to the ubiquinone (UQ) pool. Ubiquinone is reduced by the input of electrons *via* the several dehydrogenases and again oxidized by the cytochrome oxidase pathway and/or AOX. The cytochrome pathway contains the enzymes cytochrome c reductase (complex III), cytochrome c (Cyt C), and cytochrome c oxidase (complex IV, COX). Cytochrome c reductase oxidizes ubiquinone and transfers the electrons to Cyt C, which passes on the electrons to COX. This complex reduces O_2_ to two molecules of H_2_O. Due to the activity of the complexes I, III, and IV, an electrochemical proton gradient is formed across the inner membrane (**Figure 3**). The ATP synthase (complex V) uses this potential energy to generate ATP from ADP and Pi by allowing protons to flow back across the membrane down the gradient. Furthermore, plants also contain an ubiquinol-oxidizing AOX. This oxidase transfers the electrons directly to O_2_, thereby bypassing the proton pumping of the cytochrome pathway (complex II and COX). Therefore, energy is not conserved *via* this pathway and lost as heat (Millenaar and Lambers, 2003; Van Dongen et al., 2011; Päpke et al., 2014). Several studies show that AOX activity plays an important role in preventing and reducing ROS by avoiding an “over-reduction” of UQ and by maintaining the O_2_ homeostasis (Maxwell et al., 1999; McDonald and Vanlerberghe, 2006). Under normoxia, neither AOX nor COX is active at full capacity and both have been shown to compete for the distribution of electrons (Van Dongen et al., 2011; Päpke et al., 2014).

Taking together the ATP produced by the glycolysis, TCA cycle and mitochondrial electron transport chain, a total amount of 36 molecules of ATP is formed for each molecule of glucose used as substrate.

### Regulation of Central Carbon Metabolism in the Absence of O_2_ Stress

**Figure 4:**
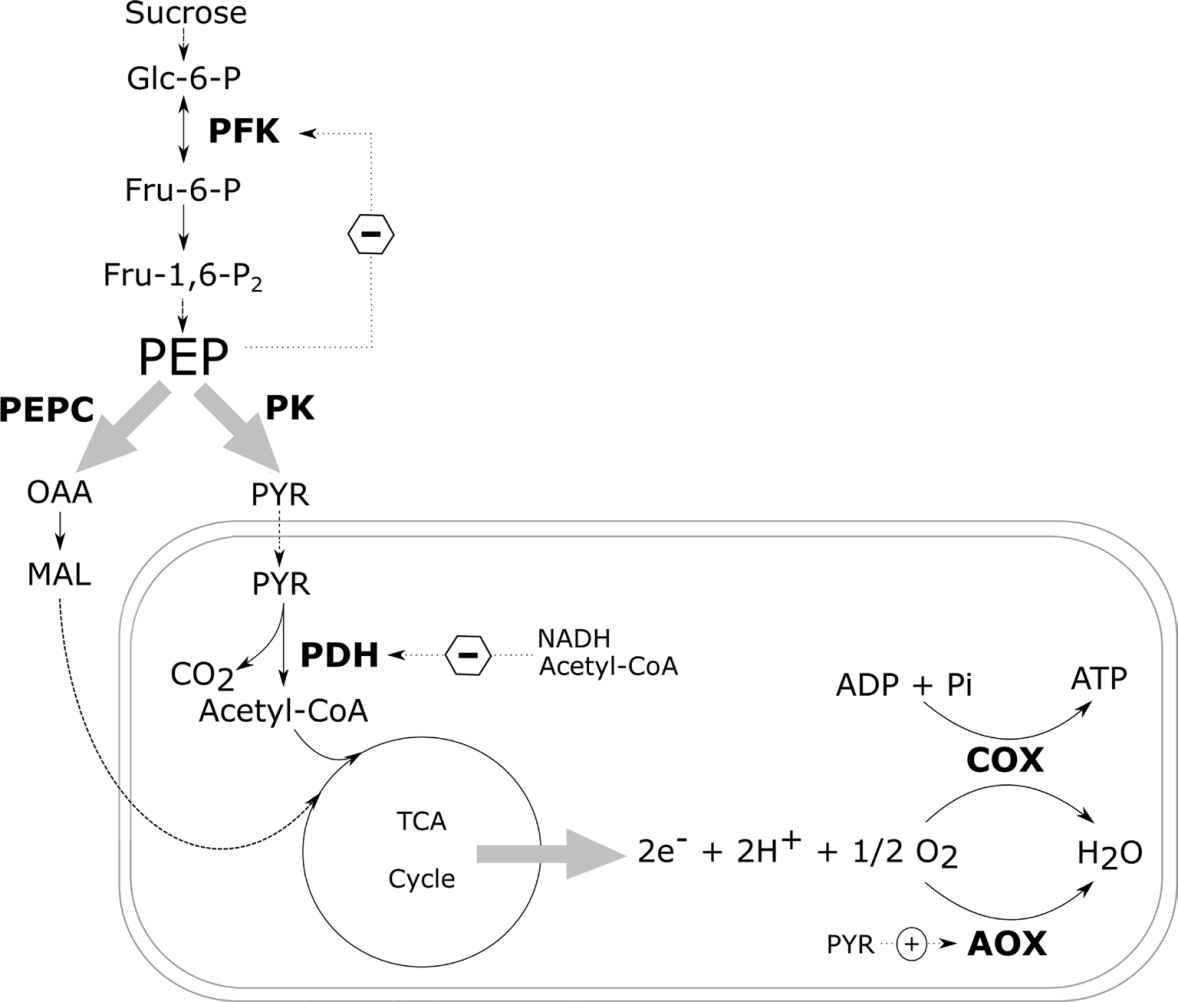
Schematic overview of the most important regulatory control sites of the carbon metabolism. The central role of PEP in the regulation of the glycolysis *via* a negative feedback mechanism is indicated, as well as the most important control sites of the mitochondrial respiration. Dotted arrows with a circled plus and minus sign indicate enzyme activation and inhibition, respectively, by allosteric effectors. Glc-6-P, glucose-6-phosphate; Fru-6-P, fructose-6-phosphate; Fru-1,6-P_2_, fructose-1,6-biphosphate; PEP, phosphoenolpyruvate; PYR, pyruvate; OAA, oxaloacetate; MAL, malate; PFK, phosphofructokinase; PEPC, phosphoenolpyruvate carboxylase; PK, pyruvate kinase; PDH, pyruvate dehydrogenase complex; COX, cytochrome c oxidase; AOX, alternative oxidase (redrawn after Plaxton and Podestá, 2006).

In nonplant systems, the glycolytic flux is regulated by phosphofructokinase (PFK), with additional control exerted by pyruvate kinase (PK). In addition, multiple studies have shown that in plants, PFK is controlled through a negative feedback from phosphoenolpyruvate (PEP) (**Figure 4**; Adams and Rowan, 1970; Kobr and Beevers, 1971; Beaudry et al., 1989; Geigenberger and Stitt, 1991; Hatzfeld and Stitt, 1991; Vanlerberghe et al., 1992; Huppe and Turpin, 1994; Paul et al., 1995; Plaxton, 1996; Plaxton and Podestá, 2006; Givan, 2007; Van Dongen et al., 2011). When the cytosolic PEP levels drop due to an increased activity of PK and PEP carboxylase (PEPC), the inhibition of PFK is lifted, thus increasing the glycolytic flux again (Plaxton and Podestá, 2006). Pyruvate kinase catalyses the final reaction of the glycolysis converting ADP and PEP to ATP and pyruvate thus also plays a critical role in the regulation of glycolysis. In spite of this, transgenic studies intervening with the glycolytic enzymes have only been able to induce minor to negligible changes in the rate of respiration suggesting that the respiratory control is unlikely to be mediated by an individual enzyme only.

**Figure 5:**
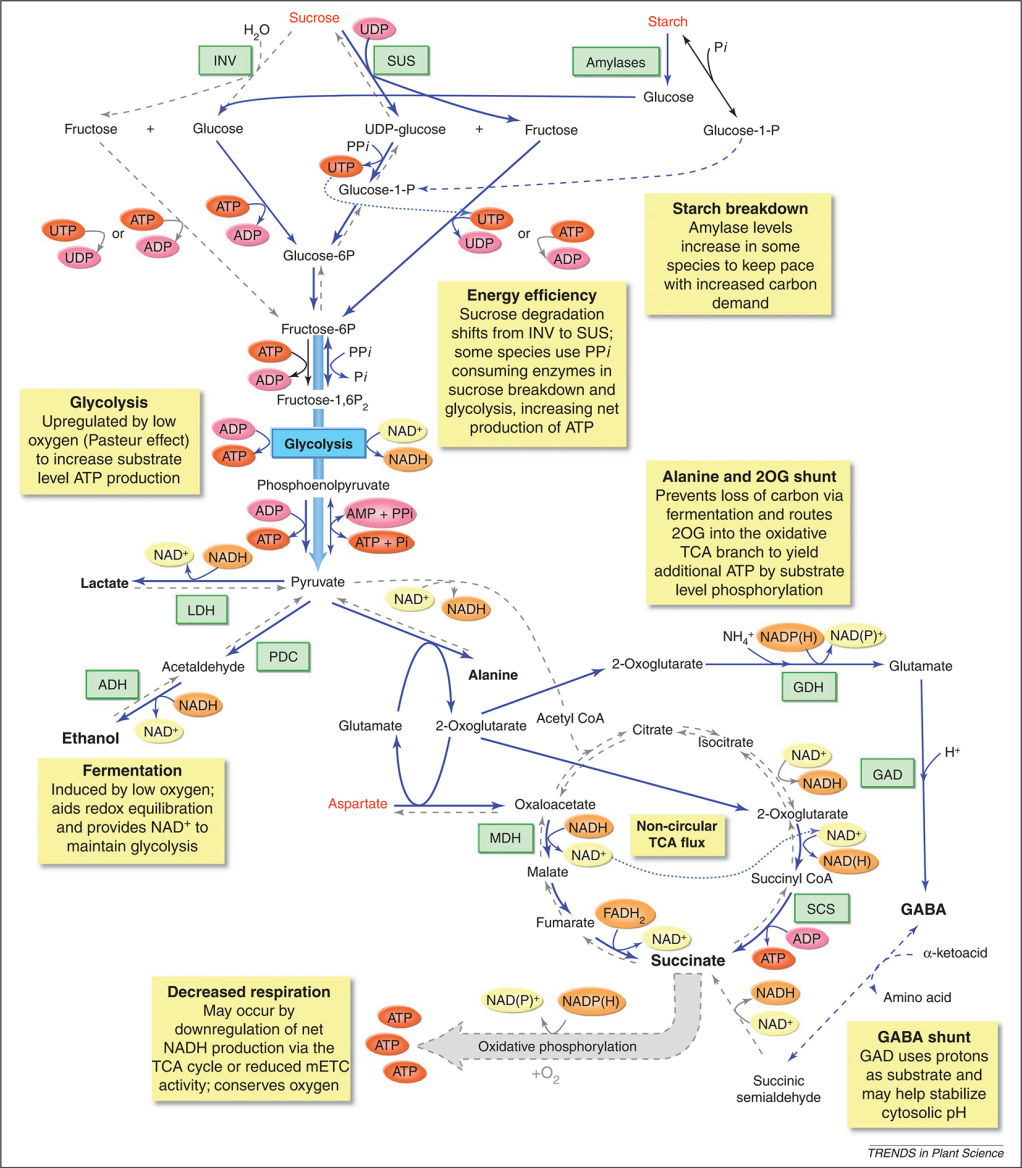
Metabolic responses of plants to low-O_2_ stress. The yellow boxes highlight a number of metabolic changes that are known to happen when O_2_ availability is limited, such as enhanced sucrose-starch metabolism, glycolysis, fermentation, a modified TCA flow, an alanine and 2-oxoglutarate (2-OG) shunt, and a GABA shunt. Blue lines indicate pathways enhanced during low-O_2_ stress, blue dashed lines indicate pathways proposed to be active during low-O_2_ stress, and grey dashed lines indicate reaction that are inhibited during low-O_2_ stress. Metabolites that increase during low-O_2_ stress are shown in enlarged black font; metabolites that decrease are shown in red font. 2OG, 2-oxoglutarate; ADH, alcohol dehydrogenase; GAD, glutamic acid decarboxylase; GDH, glutamate dehydrogenase; INV, invertase; LDH, lactate dehydrogenase; MDH, malate dehydrogenase; PDC, pyruvate decarboxylase; SCS, succinyl CoA ligase; SUS, sucrose synthase (reprinted from Bailey-Serres et al., 2012 with permission from Elsevier).

Similarly, transgenic studies focussing on the TCA cycle enzymes also only showed limited changes in respiration of photosynthetic plants (Van Dongen et al., 2011). However, in nonphotosynthetic plant tissue, respiration was clearly impacted by chemical inhibition of the TCA cycle enzyme 2-oxoglutarate dehydrogenase as was the amino acid metabolism (Araújo et al., 2008; Van der Merwe et al., 2009; Van der Merwe et al., 2010). This lack of clear impacts of changed enzyme expression on cellular respiration rate does not mean that the glycolysis and TCA cycle are not regulated at all. Both pathways are, for instance, influenced by the availability of sucrose (Fernie et al., 2002; Urbanczyk-Wochniak et al., 2006) and redox status fluctuations (Kolbe et al., 2006; Araújo et al., 2012). Furthermore, while both ATP and UTP are involved in the various steps of the glycolysis (see **Figure 5**), cellular respiration only responds to fluctuations in adenylate, but not uridylate metabolism (Regierer et al., 2002; Geigenberger et al., 2005) highlighting the central role of ATP as main cellular energy source. Altogether, these observations suggest that the mETC is pivotal to controlling respiration rate (Van Dongen et al., 2011).

The demand of ATP plays an important role in the regulation of the mETC. The activity of the mETC complexes I–IV is regulated by the proton motive force. A high cellular demand for ATP stimulates the ATP synthase complex increasing the flux of protons into the matrix. To compensate for this increase of proton influx, the activity of complex I, III, and IV is increased. In addition, the cell needs to control the production of ROS, which are a by-product of the mETC. Like mentioned in section 2.1, the mETC has several alternative electron donor and acceptor proteins, which need regulation (Van Dongen et al., 2011). While in mammals and yeast, the activity of COX can be regulated by exchanging subunits of COX (Burke and Poyton, 1998; Semenza, 2007), it is not clear whether a similar regulation exist in plants. Another regulatory mechanism might relate to the various phosphorylation sites on the mETC complexes, but for now the role phosphorylation might play is unknown (Bykova et al., 2003; Ito et al., 2009; Kadenbach et al., 2010). While the mETC is typically seen to operate in a linear way, the mitochondrial complexes can be ordered in so-called supercomplexes or respirasomes with specific configuration and stoichiometry (Eubel et al., 2004; Boekema and Braun, 2007; Schäfer et al., 2007). In plants, this is only known to occur for the complexes I, III, and IV (Schäfer et al., 2007). While the functional role of these supercomplexes is not yet clear it might increase the stability of individual complexes (Diaz et al., 2006) increasing the protein density of the membrane (Boekema and Braun, 2007). The supercomplexes might thus facilitate channelling electrons between the reactive sites, affecting respiratory rate and ATP production (Van Dongen et al., 2011; Päpke et al., 2014; Schmidt et al., 2018).

### Regulation of Central Carbon Metabolism Under Low-O_2_ Stress Conditions

#### Energy-Saving Metabolic Adaptations Upon Low-O_2_ Stress

Due to the respiratory activity and the diffusion properties of the fruit, O_2_ levels inside a pome fruit can be several factors lower than the levels of the storage atmosphere inducing low-O_2_ stress (Ho et al., 2009). This strongly affects the fruit’s metabolism. One of the most direct effects of low-O_2_ stress is the reduction of the energy status of the cells (Geigenberger, 2003). In an attempt to prevent, or at least postpone, the occurrence of internal anoxia and its concomitant negative consequences, plants try to save energy by adapting their metabolism (Geigenberger, 2003; Van Dongen et al., 2011; Bailey-Serres et al., 2012). One of these adaptations is the reduction of nonessential, energy-consuming processes such as the synthesis of storage products like starch, protein, and lipids (Geigenberger, 2003; Vigeolas et al., 2003; Van Dongen and Licausi, 2015). A second metabolic adaptation is to favor pyrophosphate (PPi) dependent reactions above those which use ATP as substrate (**Figure 5**; Bailey-Serres et al., 2012; Kosmacz and Weits, 2014).

There are at least three reactions known in the plant primary metabolism that have PPi-dependent alternative enzymes. The first known reaction involves the cleavage of sucrose. Instead of using invertases and hexokinases to provide hexose-phosphates from sucrose, which is a ATP consuming process, plants can use sucrose synthase (SUS) and UDP-glucose pyrophosphorylase (UGPase) to cleave sucrose. This alternative pathway is truly energy saving by using only one molecule PPi instead of two molecules ATP (Mustroph et al., 2014). *SUS* has been reported to be one of the core-responsive genes to hypoxia in apple fruit. The gene expression of SUS in apple fruit is highly sensitive to changes in O_2_ concentration in the environment (Cukrov et al., 2016).

Secondly, the ATP dependent glycolytic enzyme phosphofructokinase (PFK), which catalyses the phosphorylation of fructose 6-phosphate, has a reversible PPi using variant, i.e., pyrophosphate:fructose-6-phosphate phosphotransferase (PFP). However, the induction of *PFP* expression during hypoxia was not observed in all species (Mustroph et al., 2014). Interestingly, *PFK* genes were observed to be expressed during hypoxia in all plant organisms studied so far (Mustroph et al., 2010), suggesting that PFK and not PFP catalyses the main glycolytic pathway under hypoxic conditions in most organisms (Mustroph et al., 2014). A rapid upregulation of PFK expression during low-O_2_ stress was observed in apple fruit (Cukrov et al., 2016). However, how the *PFK/PFP* gene expression and activity is regulated in apple fruit still remains to be investigated.

Also, the last step of the glycolysis, where phoshoenolpyruvate is converted to pyruvate, might be catalysed by a PPi dependent enzyme; i.e., pyruvate-orthophosphate dikinase (PPDK), instead of pyruvate kinase (PK). Although these alternative pathways could compensate for the severe ATP deficiency during low-O_2_ stress, their exact impact is not yet clear. PPi is a side product produced during biosynthetic reactions, which are known to be downregulated during hypoxia. However, the content of PPi under hypoxia is reported to stay mostly stable, unlike ATP, suggesting PPi production from alternative sources. Furthermore, the alternative enzymes mostly catalyse reversible reactions, making it difficult to estimate which direction is the favorable one under low-O_2_ stress (Mustroph et al., 2014).

When plants try to save energy, the demand for respiratory O_2_ consumption decreases and the respiratory activity of the mETC slows down (**Figure 5**; Gupta et al., 2009; Zabalza et al., 2009). In this situation, plants try to make the ATP production per O_2_ that is consumed as efficient as possible. Gupta et al. (2009) observed that the ratio between the capacities of COX to AOX increases when the O_2_ availability goes down. This suggests that the amount of ATP produced by the reduction of one molecule of O_2_ will increase. Nowadays, the role of respiratory supercomplexes in the regulation of the electron flux to either AOX or COX is being investigated (Van Dongen et al., 2011). Pyruvate can bind to AOX to activate the enzyme (**Figure 4**; Oliver et al., 2008; Zabalza et al., 2009). Therefore, it seems important to control the cellular concentrations of pyruvate during low-O_2_ stress. However, in most plant species the affinity of AOX to O_2_ is one or two orders of magnitude lower compared to the affinity of COX to O_2_. Hence, it is unlikely that AOX competes with COX for O_2_ as a substrate during low-O_2_ stress (Van Dongen et al., 2011; Päpke et al., 2014).

#### Fermentative Metabolism

The goal of postharvest storage of fruit is to lower the O_2_ levels to such a degree that the respiratory metabolism is reduces as much as possible, but without inducing too many negative effects on fruit quality. One of these negative effects is the induction of fermentation. When the low-O_2_ stress induced in the tissue is too severe, the mitochondrial respiration is compromised too much, leading to insufficient supply of ATP for energy demanding processes. In an attempt to compensate for this decrease in respiratory energy production, plants increase their glycolytic flux to produce more energy with the conversion of glucose to pyruvate, a process known as the Pasteur effect (**Figure 5**).

Since apple fruit is a nonphotosynthetic organ, it needs to hydrolyse starch to provide enough sucrose to support the high glycolytic flux. The gene expression of the starch degrading enzyme, β-amylase, appears to be upregulated in “Granny Smith” apples stored under hypoxia (Cukrov et al., 2016). When the glycolytic flux increases, pyruvate starts to accumulate since it is no longer shuttled into the TCA cycle due to the decreased pyruvate dehydrogenase activity. Pyruvate dehydrogenase is inhibited by the high reduction levels of NADH/NAD^+^ pool and the accumulation of acetyl-CoA (**Figure 4**; Päpke et al., 2014). However, to be able to maintain the high glycolytic flux, NADP^+^ and NAD^+^ have to be regenerated and the accumulation of pyruvate should remain limited (Geigenberger, 2003; Bailey-Serres et al., 2012; António et al., 2016). Therefore, the increase in glycolytic flux is typically coupled to fermentation.

In the fermentation pathway, pyruvate can be converted to either lactate or ethanol and CO_2_, meanwhile maintaining the redox balance in the cell by the formation of NAD^+^ (**Figure 5**; Perata and Alpi, 1993). Pyruvate is converted to lactate by the enzyme lactate dehydrogenase. The accumulation of lactic acid will cause cytoplasmic acidosis, which can result in cell damage (Perata and Alpi, 1993). The decreasing cytosolic pH, results in a decreasing activity of lactate dehydrogenase and activation of pyruvate decarboxylase. This enzyme converts pyruvate to acetaldehyde, which is further converted to ethanol by alcohol dehydrogenase and finally to ethyl acetate by the enzyme alcohol acyl transferase. These volatile fermentation metabolites (ethanol, acetaldehyde, and ethyl acetate) readily diffuse out of the cells into the external environment, leading to a depletion of carbon reserves and causing off-flavors in the fruit (Tadege et al., 1999; Fukao and Bailey-Serres, 2004; Pesis, 2005; António et al., 2016). The accumulation of ethanol under extreme low O_2_ concentrations and/or during prolonged low-O_2_ storage has been reported for multiple apple cultivars (Mattheis et al., 1991; Saquet and Streif, 2008; Bekele et al., 2015; Cukrov et al., 2016; Brizzolara et al., 2017), also in relation to the development of low O_2_ induced physiological disorders (Vandendriessche et al., 2013; Hatoum et al., 2014; Lumpkin et al., 2014). Furthermore, it has been shown that the rate of ethanol accumulation increases with decreasing O_2_ levels (Lumpkin et al., 2014; Cukrov et al., 2016; Brizzolara et al., 2017).

Boeckx et al. (2019) performed an extensive study on the *in vivo* regulation of postharvest fermentation in apple fruit as a function of storage temperature and time. A kinetic modelling approach was used to link measured enzyme activities of PDC and ADH to the observed changes in pyruvate, acetaldehyde, ethanol, and ethyl acetate by calculating the intermediate fluxes. This revealed that control of the ethanol pathway depended on the actual conditions applied, showing both elements of molecular and metabolic control. Prolonged exposure to low-O_2_ stress resulted in a decrease in fermentation products indicating a switch to alternative pathways, possibly as an effort to minimize carbon losses (Boeckx et al., 2019).

#### Alternative Pathways Induced by Low-O_2_ Stress

Although the induction of fermentation helps apple fruit to tolerate low-O_2_ stress by mitigating the damaging effects of energy crisis, it causes acidosis of the cytoplasmic pH and depletion of the carbon reserves (Limami, 2014). Changes in amino acid metabolism may help to reduce these negative effects of fermentation. More specifically, alanine metabolism plays an important role in maintaining the glycolytic flux by converting pyruvate with the help of alanine aminotransferase (AlaAT) to alanine, providing an alternative, nondetrimental end product (**Figure 5**). Alanine is observed in a number of species, including apple (Bailey-Serres et al., 2012; Cukrov et al., 2016; Hatoum et al., 2014; Vandendriessche et al., 2013). Alanine concentrations are found to be increased in Braeburn apples after long term storage (up to 8 months) under 2.5 and 3.7 kPa CO_2_ conditions (Hatoum et al., 2014). Furthermore, it was shown that alanine accumulated to different levels depending on the composition of the atmosphere (Vandendriessche et al., 2013; Cukrov et al., 2016; Brizzolara et al., 2017). This is probably due to hypoxic stress inducing the expression of the genes coding for *AlaAT*.

Cukrov et al. (2016) showed that the accumulation of *AlaAT* transcripts was extremely abundant in apples stored under 0.4 kPa O_2_, but in fruit stored under 0.8 kPa O_2_, *AlaAT* gene expression appeared to remain at basal levels throughout the experiment. These findings suggest a low-O_2_ threshold for this gene in apple. Since the concentration of alanine was clearly higher in apples stored under 0.8 kPa O_2_ as compared to normoxia, another mode of regulation has to be present (Cukrov et al., 2016). By feeding *Medicago truncatula* (cv. Paraggio) seedlings with ^15^N-glutamate or ^15^N-alanine, Ricoult et al. (2006) provided some evidence that under hypoxia the activity of the reversible enzyme AlaAT was directed towards alanine synthesis using glutamate as amino donor while the reaction of glutamate synthesis using alanine as amino donor was inhibited. These results show that AlaAT has a dual mode of regulation by hypoxia, both at transcriptional and posttranslational level (Limami, 2014).

As can be seen from **Figure 5**, the synthesis of alanine is accompanied by the generation of 2-oxoglutarate, which can be further metabolized in the TCA cycle *via* 2-oxoglutarate dehydrogenase and succinate CoA ligase (SCS) to form succinate and produce ATP. The amount of ATP gained *via* this pathway doubles the amount of energy produced by the glycolysis alone. Succinate will accumulate as the TCA cycle is blocked due to the O_2_ limitation at the reaction catalysed by complex II of the mETC. The NAD^+^ that is required for this reaction is guaranteed by the oxidation of NADH *via* malate dehydrogenase (MDH) catalysing the reaction from oxaloacetate to malate in the reversed TCA cycle. During hypoxia, oxaloacetate is produced by aspartate aminotransferase (AspAT), simultaneously producing glutamate, which is used by AlaAT as a cosubstrate for alanine synthesis (**Figure 5**). Hence, the accumulation of alanine during low-O_2_ stress as a C/N storage compound produces extra ATP, thereby diminishing an energy crisis and saving carbon atoms, which otherwise would be lost *via* ethanol (Ricoult et al., 2006; Limami et al., 2008; Rocha et al., 2010; Bailey-Serres et al., 2012; Limami, 2014).

Furthermore, synthesis of alanine also limits cytoplasmic acidification by competing with lactate fermentation for the use of pyruvate (Ricoult et al., 2005; Ricoult et al., 2006). In addition, alanine may accumulate as a by-product of the GABA shunt, which is known to help stabilizing cytosolic pH (**Figure 5**). In the GABA shunt, glutamate is decarboxylated into GABA by glutamate decarboxylase (GDC). The formation of GABA is a proton consuming reaction, which increases the pH (Greenway and Gibbs, 2003). GABA can react further to succinic semialdehyde *via* GABA-T that uses pyruvate as amino acceptor under hypoxic conditions leading to the production of alanine (Limami, 2014). GABA content is found to be increased under low-O_2_ stress in “Granny Smith” and “Red Delicious” apples (Cukrov et al., 2016; Brizzolara et al., 2017). Cukrov et al. (2016) found a similar trend in the accumulation patterns of alanine and GABA under hypoxia, suggesting that both pathways are coregulated in apple fruit. The same authors saw a reduction of asparagine and aspartate by 0.4 kPa O_2_ but not by 0.8 kPa O_2_. This could be the result from a different modulation of the carbon flux into the TCA cycle under these two stress levels (Cukrov et al., 2016). Based on a metabolomics approach, Brizzolara et al. (2017) found that “Granny Smith” and “Red Delicious” apples have a different approach to balance the levels of pyruvate and to keep on producing energy under hypoxia. “Red Delicious” apples are producing more ethanol and GABA, while “Granny Smith” apples accumulate more alanine.

**Figure 6:**
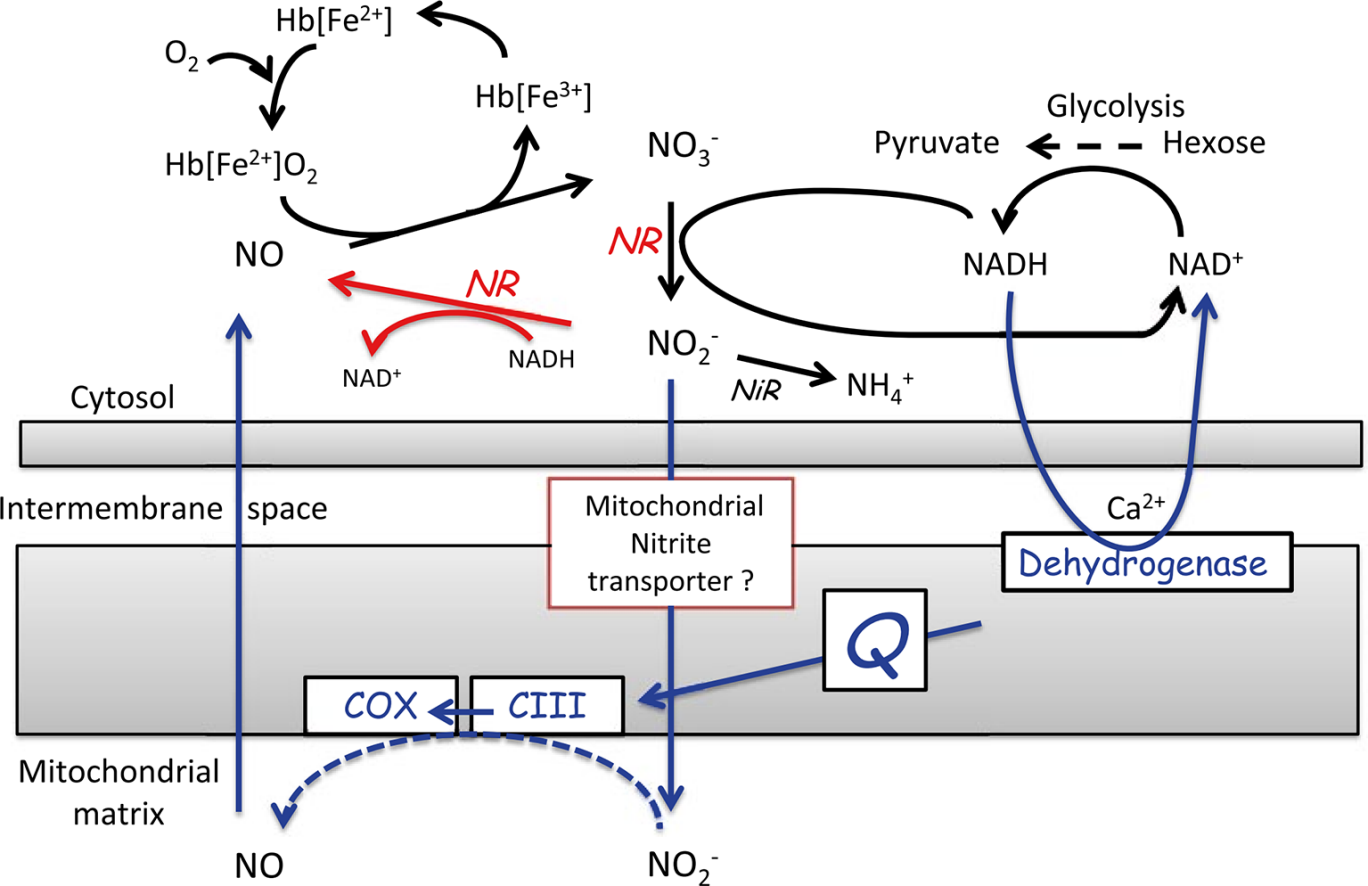
Representation of the nitric oxide (NO) cycle. NO is either produced in the cytosol by the reduction of nitrite by nitrate reductase (NR), shown in red or NO is produced in the mitochondrial matrix at levels of O_2_ below the saturation of COX, shown in blue. When NO is produced, it is assumed to be scavenged by oxyhaemoglobin [Hb(Fe_2_^+^)O_2_] to regenerate NO_3_^-^ and metHb [Hb(Fe_3_^+^)] in the NO cycle (reprinted from Limami et al., 2014. Copyright Springer-Verlag).

**Figure 7:**
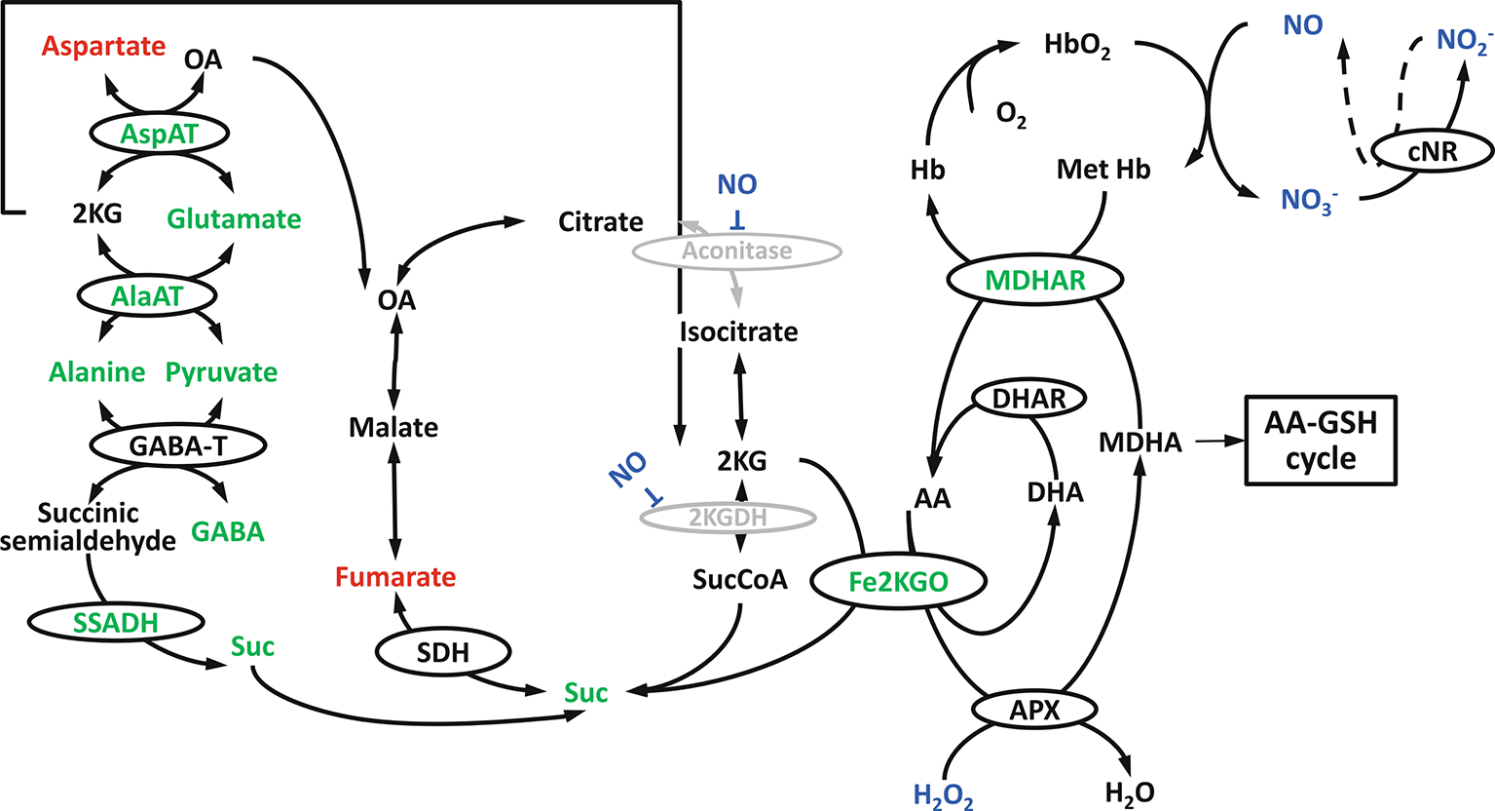
Hypoxia induced tricarboxylic acid (TCA) cycle modification in Arabidopsis shoots: the involvement of Fe2+ dependent ketoglutarate oxidase, nonsymbiotic haemoglobins, NO, alanine metabolism and GABA shunt (reprinted from Blokhina et al., 2014 Copyright Springer-Verlag).

During low-O_2_ stress, another alternative pathway is induced to recycle NAD^+^ from NADH, to be able to maintain the high glycolytic flux. This alternative pathway is the nitric oxide (NO) cycle, in which NO is either produced in the cytosol or in the mitochondrial matrix (**Figure 6**). In the latter case, nitrite is transported to the mitochondrial matrix through a yet unknown transport system, where it serves as an alternative electron acceptor at the sites of complex III and COX. The electrons necessary for this reaction are generated by the oxidation of NAD(P)H by the Ca^2+^-sensitive NAD(P)H dehydrogenase on the inner mitochondrial membrane surface. In both cases, NO is scavenged by class-1 nonsymbiotic haemoglobins (Hb), which are found to be highly expressed during low-O_2_ stress. Due to its high affinity for O_2_, Hb spontaneously oxygenate to oxyhaemoglobins, which catalyse the turnover of NO to nitrate (for further details see Igamberdiev and Hill, 2004; Limami, 2014; Limami et al., 2014; Van Dongen and Licausi, 2015). Plants can protect the cells against deleterious nitrosative stress by regulating the NO levels *via* the NO cycle. Thereby, they can control the multiple functions exerted by NO, such as the interaction of NO with hormone signalling and its inhibitory effect on haeme- and Fe-S cluster-containing enzymes such as aconitase, COX, and catalases (**Figure 7**). Through its inhibitory effect on these enzymes, NO can directly affect the energy status of the cell and their defence against ROS (Blokhina et al., 2014).

In *Arabidopsis* shoots, a metabolic association between the TCA cycle and the NO cycle under low-O_2_ stress was suggested, as can be seen in **Figure 7** (Blokhina et al., 2014). Under low-O_2_ stress, monodehydroascorbate reductase (MDHAR) can act as a MetHb reductase (Igamberdiev et al., 2006), thereby simultaneously producing ascorbate. Based on metabolomics and microarray studies under O_2_ deprivation, a novel route, besides the ascorbate-glutathione cycle, is suggested to oxidize ascorbate. In the novel route, Fe-dependent 2-ketoglutarate oxygenase (Fe2KGO) utilizes 2 ketoglutarate (2KG) and ascorbate to form succinate. Under low-O_2_ stress conditions, 2KG needed for the reaction is supplied through the metabolism of alanine. The novel route *via* Fe2KGO makes it possible to bypass the NO-inactivated TCA cycle components (Blokhina et al., 2014). Also in apple fruit, Fe2KGO genes were found to be strongly upregulated under low-O_2_ stress (Cukrov et al., 2016).

In conclusion, how well plants can tolerate low-O_2_ stress highly depends on their ability to mitigate damaging effects of energy crisis and acidosis of cytoplasmic pH (Greenway and Gibbs, 2003).

### Low-O_2_ Sensing and Signalling

Regulation of the energy metabolism in plants experiencing low-O_2_ stress demands an efficient and tuneable sensing mechanism (Kosmacz and Weits, 2014; Van Dongen and Licausi, 2015; Schmidt et al., 2018). In this section, the different molecular mechanisms plants employ to sense decreased cellular O_2_ concentrations and how they respond to this is discussed.

#### Direct O_2_ Sensing

From studies conducted on model plants like *Arabidopsis thaliana* and rice, it became clear that plants have a highly adaptive and flexible response to low O_2_, regulated by a set of cardinal genes. A set of 49 specific genes known as the hypoxia-responsive genes (HGRs) are involved in a variety of important cell adaptation processes (Mustroph et al., 2009; Mustroph et al., 2010; Lee et al., 2011; Bui et al., 2015). HRG transcript accumulation has a domino effect in facilitating an efficient, but flexible metabolic reprogramming of the cell. The up-regulation of HRGs lead to the production of pyruvate decarboxylase, alcohol dehydrogenase, lactate dehydrogenase, PFK, and alanine aminotransferase, as well as ACC synthase and ACC oxidase (Mustroph et al., 2010). These enzymes are involved in various cellular processes including carbon catabolism, anaerobic fermentation and the regulation of reactive O_2_ species (ROS) production, that help the cell adapt to the limited O_2_ available (Mustroph et al., 2009; Papdi et al., 2015). Increased ROS production due to a faltering mETC could cause oxidative damage to the cell. Therefore, upon severe hypoxia, scavenging enzymes are induced to prevent the accumulation of ROS, and protein chaperones and inhibitors of lipid peroxidation are induced to add to the defence (Pucciariello et al., 2012). Recently, some additional negative regulators of the adaptive metabolic response to low-O_2_ stress were identified, which play a role when the plant returns to normoxic conditions (Weits et al., 2014). This highlights the importance of the reversibility and timely control of the adaptive metabolic response to low-O_2_ stress (Van Dongen and Licausi, 2015). There still remains various proteins of unknown function that should be investigated further to determine their involvement and understand the pathway to its fullest (Giuntoli and Perata, 2018).

Over the past decade, research has focused on identifying a primary “O_2_ sensor” that would be responsible for the regulation of the HRG in response to lower cellular O_2_ concentrations (Gibbs et al., 2011; Licausi et al., 2011a). It was discovered that low O_2_ levels have a direct effect on the stability, and thus activity of the transcription factor subfamily from the plant specific ethylene response factor gene family. The subfamily is referred to as group-VII ethylene response factors (ERFs) and in *Arabidopsis thaliana* consist of five family members: RAP2.2, RAP2.12, RAP2.3, HRE1, and HRE2 (Nakano et al., 2006; Licausi et al., 2011b; Bailey-Serres et al., 2012; Kosmacz and Weits, 2014; Van Dongen and Licausi, 2015; Schmidt et al., 2018).

**Figure 8:**
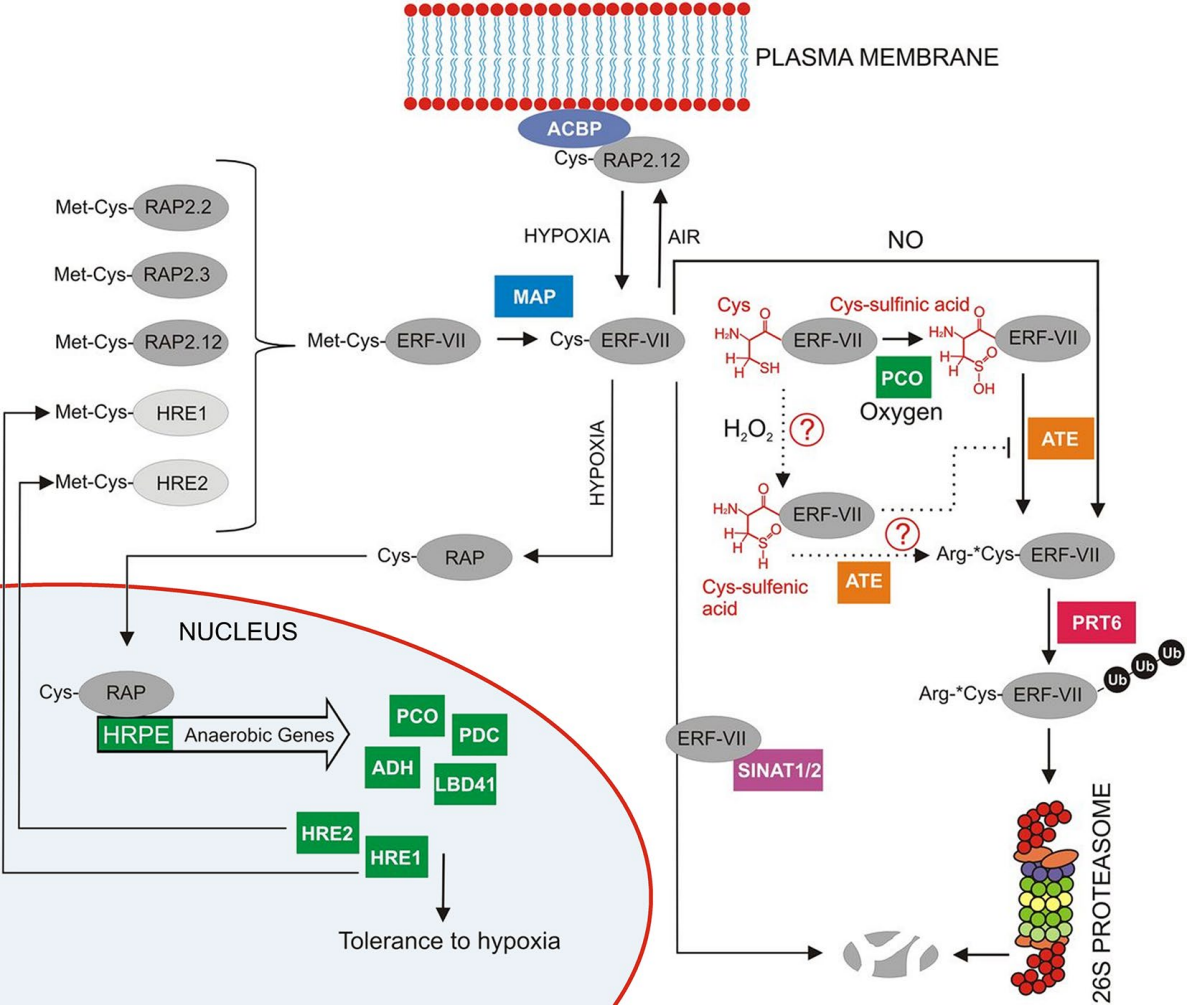
Overview of the regulation of group-VII ERF factors in Arabidopsis as reviewed by Giuntoli and Perata (2018). RAP2.12 is under normoxic conditions located at the membrane through interaction with an acyl-coenzyme A-binding protein membrane (ACBP). Any RAP2.12 dissociated from A-binding protein (ACBP) in air will eventually be degraded by the 26S proteasome after initial Met cleavage through MAP (methionine aminopeptidase). The subsequently exposed N-terminal Cys is susceptible to oxidation through plant Cys oxidases (PCO). Also, NO can play a role in this N-end rule pathway with E3 ubiquitin ligase responsible for labelling the substrate for final degradation. When O_2_ availability becomes limited, RAP2.12 no longer gets oxidised by the N-end rule pathway and is able to move to the nucleus to trigger the transcription of hypoxia responsive genes (reprinted with slight modification from Giuntoli and Perata, 2018, Copyright American Society of Plant Biologists).

The protein levels of ERF-VII transcription factors are regulated through an ancient, conserved branch of the ubiquitin proteasome system known as the N-end rule pathway (**Figure 8**), which acts as a safeguard mechanism that continuously targets the ERF-VII transcription factors for proteolysis under aerobic conditions (Gibbs et al., 2016). The N-end rule pathway regulated through a highly conserved N-terminal domain was first described by Nakano et al, in 2006. The domain is a 5-amino acid motif initiated with Methionine (Met) and Cysteine (Cys) residues. It was found to be specifically conserved in flowering plants. Ultimately, the domain acts as an N-degron, shuttling the protein into the N-end rule pathway and targeting it for proteolysis (Gibbs et al., 2011; Licausi et al., 2011b; Giuntoli and Perata, 2018).

It is known that the Cys2 from the motif has a highly reactive thiol group attached, which is sensitive to O_2_. The Cys2 is therefore known as a regulatory Cys (White et al., 2017). When the initial methionine is removed from the N-terminus *via* methionine aminopeptidase (MAP), it exposes the Cys2 to the activity of plant cysteine oxidases (PCOs). In the presence of O_2_ and NO, PCO oxidizes the Cys2 to sulfinic or sulfonic acid (Weits et al., 2014; White et al., 2017; Giuntoli and Perata, 2018), which acts as an acceptor for the condensation of an arginine residue by arginyltransferase (ATE). The exposed arginine residue is subsequently ubiquitinated by the proteolysis (PRT) E3 ligases and targeted to the 26S proteasome for degradation (Kosmacz and Weits, 2014; Van Dongen and Licausi, 2015; Schmidt et al., 2018). Therefore, this mechanism provides an efficient way to regulate the metabolic response under low-O_2_ stress conditions; the presence of O_2_ and NO causes ERF-VII destabilization, whereas their absence permits stabilization and accumulation of ERF-VII transcription factors in the nucleus (Gibbs et al., 2011; Licausi et al., 2011a; Van Dongen and Licausi, 2015; Schmidt et al., 2018). However, it is still unclear how exactly O_2_ and NO act together to oxidize ERF-VII (Van Dongen and Licausi, 2015).

What has, however, become clearer is the specific oxidization method PCO proteins employ in oxidizing the cysteine residue of the N-degron. Research recently identified various characteristics of the *Arabidopsis* PCO proteins that allow them to biochemically sense the presence of molecular O_2_ and efficiently oxidize Cys2 (White et al., 2018). After verifying the sensing capabilities of the protein, it was proposed that the PCO’s act as O_2_ sensor, instead of the previously speculated RAP2.12. The PCOs were shown to effectively regulate ERF-VII protein levels and directly influence protein stability. A direct connection between ERF-VII protein levels and PCO transcript levels could be made where it would seem that a negative feedback loop exists between RAP2.12 and PCOs. RAP2.12 is directly responsible for the up-regulation of *AtPCO*s. Increased levels of PCO proteins, consequently, lead to the active oxidation of and breakdown of ERV-II proteins *via* the N-end rule. It prevents the unnecessary buildup of the ERF-VII proteins in the nucleus. These findings open up new doors for gene manipulation and the potential to engineer increased low-O_2_ stress tolerance in plants (White et al., 2017; White et al., 2018).

For the most part, research on low-O_2_stress in plants has mainly focused on the ERF-VII transcription factor RAP2.12, specifically *AtRAP2.12*. It was found to be localized at the plasma membrane under aerobic conditions (Licausi et al., 2011a) through interactions with the peripheral membrane proteins acyl-coenzyme A-binding protein 1 (ACBP1) and ACBP2 (**Figure 8**). This interaction protects the RAP2.12 protein from being shuttled into the N-end rule pathway for degradation. However, under hypoxic conditions, RAP2.12 can detach from the membrane and is transported to the nucleus where it then accumulates. The protein accumulation subsequently triggers the expression of the 49 HRGs. It has been speculated that the accumulation of RAP2.12 at the plasma membrane under normoxic conditions might be a reservoir of activators, which enables a quick induction of the HRGs and an efficient response to declining cellular O_2_ levels (Kosmacz and Weits, 2014; Van Dongen and Licausi, 2015). As seen with the recent PCO-based discoveries, much about how RAP2.12 is regulated during low-O_2_ stress is still unknown.

When referring to regulation of the low-O_2_ stress, it also entails adapting to and managing O_2_ concentration fluctuations. Plant cells should be able to adapt quickly and efficiently not only to hypoxic conditions, but also to a sudden return to normoxic conditions. When O_2_ becomes available again, it is unlikely that the displacement of the ERF-VII proteins from the promotors of the hypoxia responsive genes and their subsequent degradation is sufficient to rapidly silence the expression of the core HRGs (Van Dongen and Licausi, 2015). In *Arabidopsis*, it was found that a transcriptional regulator, hypoxia response attenuator 1 (HRA1) was responsible for the repression of the upregulated HRGs by binding to RAP2.12 (Giuntoli et al., 2014). Since RAP2.12 induces the expression of HRA1, as is the case with PCO, it acts as a positive regulator while PCOs and HRA1 restrict the function of RAP2.12 in a feedback loop, depending on the availability of O_2_ (Weits et al., 2014).

The contribution of each individual group-VII ERF to sensing low-O_2_ stress has remained largely unknown for the remaining 4 family members (Gasch et al., 2015). Transgenic studies conducted in RAP2.12, RAP2.2 and RAP2.3 overexpressing lines indicated that all three genes are responsible for activating the important set of 49 HRGs (Papdi et al., 2015). Both RAP2.12 and RAP2.2 were responsible for gene transactivation when studied in protoplasts, indicating a redundant role (Licausi et al., 2011b; Weits et al., 2014; Papdi et al., 2015). This type of redundant transactivation of similar target genes is not uncommon for plant transcription factors and was confirmed through promoter studies. It was shown that both RAP2.12 and RAP2.2 are cardinal to the plants stress response. Both transcription factors are able to recognize and bind to a conserved 12 base pair domain that acts as a *cis*-regulatory motif for the transactivation of HRGs. Initial studies also opened up speculation surrounding the remaining two family members HYPOXIA RESPONSIVE ERF1 and 2 (HRE1 and HRE2) (Gibbs et al., 2011; Licausi et al., 2011b). Subsequent research pointed to the idea that they play minor, supportive roles in the response mechanism (Papdi et al., 2015). However, Gibbs et al. (2011) showed that not all hypoxia responsive genes are controlled by the N-end rule pathway, giving the first indication that alternative regulatory pathways should exist. Further research is still required to discover more of the potential role the additional family members might play.

In an early study of the apple genome, eight genes were assigned as coding for ERF-VII transcription factors (Girardi et al., 2013), while the first evidence of an O_2_ sensing mechanism based on the N-end rule pathway and on the posttranslational regulation of ERF-VII protein stability in apple fruit is given by Cukrov et al. (2016). Unfortunately very little research has focused on nonmodel plants and further research is necessary to fully understand the behavior of the O_2_ sensing mechanism in apple fruit and its effect on the different metabolic responses upon low-O_2_ stress between the different apple varieties.

#### Discovering the Multifunctional Role of Group-VII ERFS

As research in the field expands, more evidence of additional regulatory roles for group-VII ERFs are being discovered. It would seem that the influence and regulatory function of this subfamily expands to processes related to germination, abiotic stress tolerance, and an increased resistance against pathogen infection (Giuntoli and Perata, 2018). It is hypothesized that the additional physiological roles the group-VII ERFs take on, are mainly facilitated by the occurrences of hypoxic microenvironments throughout plant tissue.

Low-O_2_-associated secondary signalling pathway has been investigated intensively where it was found that when respiration is affected by low-O_2_ stress, it has an impact on many parameters and processes, including reactive O_2_ and nitrogen species (ROS/RNS) homeostasis, redox status of NAD(P)H and antioxidant pools, ATP/ADP ratio, the proton-motive force, calcium, and metabolites levels, which could all trigger the mitochondrial retrograde responses (Schmidt et al., 2018; Wagner et al., 2018). It became clear that the RAP-type group-VII ERF genes are directly involved in and actively participate in both osmotic – and oxidative stress tolerance (Papdi et al., 2015). Studies showed that the group-VII ERF induced signalling could contribute to transcriptional reprogramming of the nuclei of hypoxic cells and is commonly referred to as the mitochondrial retrograde regulation. For a more detailed discussion on the role of these diverse signals in mitochondrial retrograde responses and its role in the low-O_2_ response of plants, the reader is referred to the review of Wagner et al. (2018).

## Perspectives and Future Challenges

Although much is known about the regulation of the central carbon metabolism in response to low- O_2_ stress in plant systems in general, many questions still remain to be answered, especially regarding apple fruit. The regulation of the carbon metabolism was already shown to differ between apple cultivars. In addition, due to the ongoing ripening of apple fruit during prolonged storage, the contribution of the various regulatory events are likely to change during the lifetime of an apple (Beshir et al., 2017).

Some aspects have not been studied yet in full for any particular plant system, such as the function of the supercomplexes and the regulation of the COX of the mitochondrial electron transport chain. While the PPP recently received quite some attention because of its role in retaining redox homeostasis in human diseases (Stincone et al., 2015), its role during low-O_2_ stress conditions in apple fruit can only be assumed for now.

Even though the apple genome has been sequenced, the bottle neck remains the quality of the annotation (Velasco et al., 2010; Daccord et al., 2017). This makes it difficult to confirm findings on the molecular regulation found in other plant species in apple. The added challenge is that many of the commercial apple cultivars are polyploid not only making the linking of genotype to phenotype an even more challenging task, but also hindering assembling of the true biological genome from sequencing data (Kyriakidou et al., 2018).

To study the molecular control in response to low-O_2_ stress, *in vivo* experiments under well controlled O_2_ conditions need to be designed. In bulky organs like apples, fruit internal O_2_ gradients will inevitably arise complicating interpretation of the experimental results using intact fruit. To this end, integrated mathematical models are urgently needed that combine knowledge on the physics of gas transport with expertise on molecular and metabolic control of the central carbon metabolism in apple (Ho et al., 2009; Ho et al., 2011; Ho et al., 2018).

Recently, exciting research using classical model plant systems has discovered a mechanism of O_2_ sensing in plants. This work bears large relevance to the postharvest physiology of apple fruit exposed to low-O_2_ storage conditions. It, therefore, is important to fully understand the behavior of apple fruit exposed to low-O_2_ stress to ultimately minimise the incidence of low-O_2_ related storage disorders, not just by trial and error, but through a proper understanding of the regulation of their central carbon metabolism.

## Author Contributions

JB wrote the first draft of the manuscript. SP and MH wrote sections of the manuscript. All authors contributed to manuscript revision, read and approved the submitted version.

## Conflict of Interest

The authors declare that the research was conducted in the absence of any commercial or financial relationships that could be construed as a potential conflict of interest.
